# Comments on “Notes Relating to the Extirpation of the Parotid Gland,” by Prof. Brainard, M. D. Remarks on the Surgical Treatment of Spina Bifida—Summary of Prof. Nelaton’s Clinical Lecture on the Injection of Solution of Iodine in Cases of Hydrorachitis, after the Manner Employed by Prof. Brainard

**Published:** 1861-06

**Authors:** Titus Deville


### ARTICLE II.

COMMENTS *on "Notes relating to the Extirpation of the Parotid Gland," by Prof. Brainard, M. D., in the Chicago Medical Journal for October*, 1860.

REMARKS *on the Surgical Treatment of Spina Bifida—Summary of Prof. Nelaton's Clinical Lecture on the Injection of Solution of Iodine in cases of Hydrorachitis, after the Manner employed by Prof. Brainard.*

---

By TITUS DEVILLE, M. D.

---

We suppose we must consider these notes of Prof. Brainard as a sort of rejoinder to our critical essay published in the *Chicago Medical Examiner*, but they are really so weak and so little is advanced in them in reply to the arguments then brought forward, that we conclude therefrom that Prof. Brainard is utterly incapable of meeting them with powerful opposing facts deduced from the sound knowledge of anatomy and pathology, and the experience of the best modern surgeons. We are obliged, therefore, to content ourselves with combatting the sophistry so easily detected in these "notes," and refer your readers to the essay in question, to enable them more fully to arrive at a correct judgment of the real merits and objects of the discussion.

Prof. Brainard cites the opinions of Professors Granville, Sharp, Patteson, Mutter, Vidal and Gross, on the *possibility* of the extirpation of the Parotid. Now we here protest against the inference with which Prof. Brainard would seemingly wish to blind his readers: That the possibility of the extirpation of the Parotid had been called into question by us. We did not assert the utter impossibility of its removal; but contended that it was a dangerous operation and not easy of accomplishment, in contradiction to the opinion of Prof. Brainard. We advanced the opinion that the Parotid was remarkably exempt from malignant disease; when affected, we believe it is from disease being propagated to it from other structures.

According to Prof. Brainard, this gross error into which we have fallen, ought at once to be detected by any medical practitioner, and yet we find that Velpeau, Ferguson and Liston, the great practical authorities on which such an opinion was founded, emphatically deny that they have ever seen disease of the Parotid; that what was supposed to be disease of the Parotid, was not in reality such, but only of one or more of the lymphatic glands in this region, and which were comparatively easy to remove and unattended with any great danger from being limited by a cellular cyst.

In addition to the strong testimony on this point given by the three greatest surgeons of modern times, we have also the negative evidence of the most recent writers on pathology, such as Houel, Wilks and Bennett, who do not cite one case of disease of the Parotid (excepting enchondroma, which is a rare malady,) which, coupled with the non-existence of real specimens in some of the greatest museums in Europe, must make any intelligent medical man hesitate before he receives the number of ninety-one cases, compiled by Prof. Brainard as bearing the slightest impress of truth, and which cases are to be considered in no other light than as so many mistakes as to the real nature of the malady. It becomes totally another question when the disease is not limited to the lymphatic glands; in such a case we find all the neighboring parts more or less involved; we cannot then call it *cancer of the Parotid*; the propagation to structures on the side of the neck, lower jaw, and deep-seated parts, prevent such a denomination. Most medical men have seen such cases.

Moreover, to prove from another point of view, the proneness of the lymphatic glands to cancerous disease, and the exemption of the salivary glands, we may cite the submaxillary gland, *which in ultimate structure is like that of the Parotid*, and we fearlessly avow that we have never heard or read of a case of cancer of this gland by any notable surgeon—notwithstanding that we have searched innumerable authorities on this question.

Dr. Paoli referred to in the "Notes," has compiled from a great number of old authorities, cases of supposed cancer and extirpation of the Parotid; as well might he cite testimony, respectable in its day and generation, in favor of any other exploded error; it is only in recent times, that we have been enabled to determine with precision, the nature of those maladies. These remarks apply equally to the cases cited by Prof. Brainard.

We are somewhat surprised and amused at the unreserved manner with which Prof. Brainard cites Prof. Gross as an authority, when it may serve his purpose, recollecting the grave doubts which he expresses on Prof. Gross being considered as perfectly reliable, in a critical review of the learned Professor's work on pathology, in the very number of the *Chicago Medical Journal* for 1859, in which he narrates the cases of supposed extirpation of the Parotid. Finally, the main question which we sustained, seems to have been entirely overlooked by Prof. Brainard, viz: that we contested the interpretation which he put on the operations performed by him in the Parotid region, and that from the description given, we were convinced he had fallen into error on their real nature. We perfectly agree with him that what he *really effected was safe and practicable*, but what he *proposed to do* was very dangerous, and that *he did not remove the Parotid*, because it was not involved in the cases so described.

We here terminate our comments on "*Notes Relating to the Extirpation of the Parotid Gland*," and devote the second part of our communication to a few brief remarks on the injection of solutions of Iodine in cases of Hydrorachitis, after the manner employed by Prof. Brainard.

We are tempted to this step, and believe it will prove interesting to your readers in consequence of some critical remarks made by Prof. Nelaton, at the Clinical Hospital of the Faculty of Medicine, Paris, in his clinical lecture delivered on the 22d of March last.

Having been apprized beforehand that two cases would be commented upon by Prof. Nelaton, in which he had injected solutions of Iodine, we were anxious to learn the opinion of one of the most enlightened surgeons of modern times on such an operation; premising that we were averse to any surgical interference in such cases, further than the mere protection of the tumor, from having had, ourself, considerable practical experience of the evils resulting from various plans which had for their object the disappearance of the tumor.

Theoretically and practically, we do not hesitate to say that in the vast majority of cases active surgical interference is unadvisable; but before we enter into our reasons for such an opinion, let us briefly define the nature of the lesion.

Spina bifida is an arrest of development, in which a portion of one or more of the neural arches is wanting; viz: the laminæ and spines of one or several vertebræ, usually situated in the lumbar and sacral regions, but occasionally found in other parts of the vertebral column. Over this region, where the spine is cleft, exists a fluctuating tumor from an abundance of the cerebro-spinal fluid which is there collected; because the membranes enveloping the spinal cord being deprived of the support naturally given by the neural arches, bulge out the skin, and fluid is accumulated, forming a tumor along the middle line of the back.

*Reasons, Theoretical and Practical, against Active Surgical Interference.*

1st.—The fluid, which forms the great bulk of the tumor, has a conservative action, tending to diminish greatly the effects of any blow or fall on the tumor, (which, when situated in the lower part of the vertebral column usually contains the terminal part of the spinal chord and its nerves,) by equalizing the pressure and preventing the mischief which would ensue from any direct violence to the nervous centres and the nerves which emanate therefrom.

2d.—That any evacuation or absorption of this fluid, whether by means of a fine seton, or by the injection of an irritating solution, exposes the cord and nerves to *immediate danger* from inflammatory action, paralysis, convulsive movements, or other disturbance of the nervous functions, together with their *remote mischievous results.*

3d.—Most surgeons know of examples in which these tumors on being left to themselves do not cause any constitutional symptoms requiring surgical treatment; but persons may and do live the ordinary term of life without suffering any inconvenience therefrom, further than by their bulk.

4th.—Most surgeons know that death frequently ensues from actively interfering with such tumors,—for our own part, we have seen upwards of twenty cases terminate fatally.

To sum up briefly the remarks of Prof. Nelaton, in the lecture referred to, he treated successively of the

*Principles of the Operation.*—Absorption of the fluid contained in the tumor, thus diminishing the bulk or causing its total disappearance.

*Cases to which it is Applicable.*—A very small number, where the tumor is pediculated, and the communication can be interrupted between the sac of the tumor and the cavity of the spinal canal, so that the fluid injected shall not pass along the membranes of the cord, and also where the cord is not placed within the tumor. He cited a case of M. Robert, of the Hotel Dieu, in which, whilst injecting such a tumor, the *child died immediately;* at the autopsy the injected fluid was found to have mounted as *high as the medulla oblongata!*

*Mode of Procedure.*—Great precaution necessary; he detailed the method with the formula employed by Prof. Brainard, showing that the quantity of fluid to be injected should be small, and that the iodine solution should be very weak.

*Results and Deductions.*—Paraplegia in both cases. He confessed it had been a very unsatisfactory method in his hands; two children previously in a good state of health suddenly rendered paraplegic.—Without positively denying the truth of Prof. Brainard's results, he yet expressed some doubts as to the correctness of all the cases reported.

